# Validation of Suitable Reference Genes for Expression Normalization in *Echinococcus* spp. Larval Stages

**DOI:** 10.1371/journal.pone.0102228

**Published:** 2014-07-11

**Authors:** Sergio Martin Espínola, Henrique Bunselmeyer Ferreira, Arnaldo Zaha

**Affiliations:** 1 Programa de Pós-Graduação em Genética e Biologia Molecular, Universidade Federal do Rio Grande do Sul, Porto Alegre, Rio Grande do Sul, Brazil; 2 Departamento de Biologia Molecular e Biotecnologia, Instituto de Biociências, and Centro de Biotecnologia, Universidade Federal do Rio Grande do Sul, Porto Alegre, Rio Grande do Sul, Brazil; Nazarbayev University, Kazakhstan

## Abstract

In recent years, a significant amount of sequence data (both genomic and transcriptomic) for *Echinococcus* spp. has been published, thereby facilitating the analysis of genes expressed during a specific stage or involved in parasite development. To perform a suitable gene expression quantification analysis, the use of validated reference genes is strongly recommended. Thus, the aim of this work was to identify suitable reference genes to allow reliable expression normalization for genes of interest in *Echinococcus granulosus* sensu stricto (s.s.) (G1) and *Echinococcus ortleppi* upon induction of the early pre-adult development. Untreated protoscoleces (PS) and pepsin-treated protoscoleces (PSP) from *E. granulosus* s.s. (G1) and *E. ortleppi* metacestode were used. The gene expression stability of eleven candidate reference genes (*βTUB*, *NDUFV2*, *RPL13*, *TBP*, *CYP-1*, *RPII*, *EF*-*1α*, *βACT-1*, *GAPDH*, *ETIF4A-III* and *MAPK3*) was assessed using geNorm, Normfinder, and RefFinder. Our qPCR data showed a good correlation with the recently published RNA-seq data. Regarding expression stability, *EF-1α* and *TBP* were the most stable genes for both species. Interestingly, *βACT-1* (the most commonly used reference gene), and *GAPDH* and *ETIF4A-III* (previously identified as housekeeping genes) did not behave stably in our assay conditions. We propose the use of *EF-1α* as a reference gene for studies involving gene expression analysis in both PS and PSP experimental conditions for *E. granulosus* s.s. and *E. ortleppi*. To demonstrate its applicability, *EF-1α* was used as a normalizer gene in the relative quantification of transcripts from genes coding for antigen B subunits. The same *EF-1α* reference gene may be used in studies with other *Echinococcus* sensu lato species. This report validates suitable reference genes for species of class Cestoda, phylum Platyhelminthes, thus providing a foundation for further validation in other epidemiologically important cestode species, such as those from the *Taenia* genus.

## Introduction

Echinococcosis, one of the 17 neglected tropical diseases prioritized by WHO [1], is caused by species from the genus *Echinococcus*. According to the latest revision [2], this genus includes nine species, two of which have medical and public health relevance in humans: *Echinococcus granulosus* sensu lato (s.l.), which is the causative agent of cystic echinococcosis (CE), and *Echinococcus multilocularis*, which is responsible for alveolar echinococcosis (AE). Previously, the complex *E. granulosus* was represented by several genotypes or strains (G1–G10) of the same species [3], [4]. Currently, *E. granulosus* s.l. includes five independent species as follows: *E. granulosus* sensu stricto (s.s.) (G1–G3), which is responsible for the majority of human CE, and *E. equinus* (G4), *E. ortleppi* (G5), *E. canadensis* (G6–G10), and *E. felidis*, which are responsible for fewer human CE cases [2], [5]. CE results in a loss of 1–3 million disability-adjusted life years (DALYs) per annum, and it shows a cosmopolite distribution, having a hyperendemic pattern in several regions of East Africa, Central Asia, China, and South America (including Argentina, Uruguay, Chile, Peru, and Southern Brazil). In these areas, a CE prevalence of 20–90% is observed in slaughtered animals, leading to annual losses of approximately US$ 3 billion [1].

The life cycle of *Echinococcus* species encompasses two different hosts as follows: the intermediate host (generally domestic ungulates) where the infective larval stage (metacestode) occurs, resulting in protoscoleces, which is the pre-adult parasite form, and the definitive host (usually canids) where the differentiation of protoscoleces to the adult form occurs. Through sexual reproduction, the adult form generates eggs that, once eliminated by the definitive host feces, are ingested by the intermediate host, thereby completing the cycle. In *E. granulosus* s.l. species, the metacestode stage is represented by the formation of a unilocular hydatid cyst, filled with hydatid fluid, and more internally by a germinal layer, which gives rise to protoscoleces [6], [7]. A particular developmental characteristic of protoscoleces is the possibility to re-differentiate into secondary cysts or microcysts [6], which naturally occurs by hydatid cyst rupture and release of its content in the intermediate host, with each protoscolex being able to generate an individual secondary cyst. The ability to infect and survive in a wide range of intermediate hosts and the peculiar bidirectional development features make the *Echinococcus* species an interesting model to study host-parasite relationships and parasite development. The high impact of this disease on livestock and public health also highlights the necessity of basic studies on *Echinococcus* spp. to identify molecular targets and develop new strategies for CE control and eradication.

In recent years, a significant amount of *Echinococcus* spp. sequence data (both genomic and transcriptomic) have been published [8]–[10], facilitating the analysis of genes differentially expressed in specific life cycle stages or potentially involved developmental events of the parasite. Several upregulated or downregulated genes have been recently described in a transcriptomic survey of *E. granulosus* [8].

The microarrays and the RNA-seq are some of the several methodologies to detect different gene expression levels, and commonly are used to have a global vision of the gene expression. Moreover, when the focus is a limited number of target genes, the quantitative PCR (qPCR) is the gold standard method to detect the gene expression variations of a specific mRNA [11]. Comparing to conventional methods of quantification, such as the RNase protection assay or northern blot, the qPCR have the advantage of high sensitivity, specificity, reproducibility, and broad dynamic range, making it one of the most widespread techniques in many areas of research [12], [13].

To perform a suitable and reliable gene expression quantification analysis, reference genes need to be validated. Reference (or normalizer) genes are defined as those with a stable expression under previously defined conditions, thus appropriate to quantify gene expression levels of specific targets. To date, there are no reports on the validation of reference genes for members of the class Cestoda, phylum Platyhelminthes. There have been several reports on the identification of housekeeping genes for the larval infective stage of *E. granulosus* [14]–[18]. Furthermore, because the widely used *βACT-1* gene is upregulated in immature adults of *E. granulosus* and is variably expressed in the other developmental stages [19], the eukaryotic translation initiation factor (*Eg-eif*) has been proposed as an alternative housekeeping gene. Without previous validation of the reference gene, the accuracy of gene expression data obtained by high sensitivity methodologies, such as qPCR, will be directly affected.

In this study, we describe the identification of a suitable and reliable reference gene for normalizing the expression of specific target genes in *E. granulosus* s.s. and *E. ortleppi* protoscoleces upon induction of early pre-adult development by pepsin treatment. Using the validated reference gene, we quantified the relative mRNA expression of differential and constitutive expressed genes between both PS and PSP conditions, as previously described [10], [20]. Finally, we analyze the expression of the *E. granulosus* genes coding for antigen B (EgAgB) subunits and compare with previous results of RNA-seq and qPCR data [8], [19], [21].

## Materials and Methods

### Sample collection, treatments and genotyping

Bovine hydatid cysts were obtained from the Cooperleo Abattoir (São Leopoldo, Rio Grande do Sul, Brazil). The slaughtered animals came from different regions of the Rio Grande do Sul, mostly from farms located in the south and southwest of the state. The protoscoleces were collected by hydatid cyst fluid aspiration and washed at least five times with 1× phosphate buffered saline (PBS). Viability was assessed through optical microscope observation and trypan blue staining. Only protoscoleces with viability greater than 90% were used for further analysis. For species determination, a high-resolution melting (HRM) genotyping method was performed using part of the cytochrome c oxidase subunit I (*cox1*) gene, which has distinct melting curves that allow discrimination between *E. granulosus* s.s. (G1) and *E. ortleppi* [22].

Focusing on the early development of the pre-adult form, we used two different conditions: 1) protoscoleces were directly extracted from hydatid cyst and washed with 1× PBS (PS) and 2) protoscoleces were treated with pepsin (PSP). For the PSP group, after washing with 1× PBS, the samples were treated with pepsin (2 mg/mL) for 15–20 min at pH 2 to mimic the contact with the digestive enzymes of the definitive host, thus achieving an “activated” or development-induced state [6]. After pepsin treatment, the evagination of protoscoleces and their flame cell movements were clearly evident by microscope observation.

### Total RNA extraction and cDNA synthesis

PS or PSP samples (50–100 µL, containing approximately 5000–10000 individuals) were mixed with 1 mL of TRIzol reagent and immediately frozen in liquid nitrogen until the total RNA extraction. Total RNA was isolated using TRIzol reagent in conjunction with the PureLink RNA Mini Kit according to the manufacturer's protocol. After treatment with RNase-free DNase I (Thermo SCIENTIFIC) for 30 min at 37°C to remove all genomic DNA, total RNA concentration was determined using a Nanodrop ND2000 spectrophotometer (Thermo SCIENTIFIC). Quality and integrity were assessed by 1.5% agarose gel electrophoresis and by an Agilent 2100 Bioanalyzer using an RNA 6000 Pico Chip Kit. The first strand of cDNA was synthesized from 100 ng of total RNA using RevertAid reverse transcriptase (Thermo SCIENTIFIC) and Oligo (dT)_18_ (0.5 µg/µL) as the anchor primer. The reaction mixture was incubated at 42°C for 1 h followed by 70°C for 10 min to terminate the reaction and brought to a final volume of 20 µL. The final cDNA product was diluted 50-fold with nuclease-free water prior to use in qPCR analysis.

### qPCR analysis

Eleven genes were selected for expression studies, namely beta tubulin (*βTUB*), NADH dehydrogenase ubiquinone flavoprotein 2 (*NDUFV2*), L13 ribosomal protein (*RPL13*), TATA-box binding protein (*TBP*), cyclophilin 1 (*CYP-1*), RNA polymerase II subunit RPB2 (*RPII*), elongation factor 1 alpha (*EF-1α*), beta actin 1 (*βACT-1*), glyceraldehyde-3-phosphate dehydrogenase (*GAPDH*), eukaryotic translation initiation factor 4A-III (*ETIF4A-III*) and mitogen activated protein kinase 3 (*MAPK3*) genes. Gene sequences obtained from different databases (LophDB, GeneDB, and GenBank) were used to design specific primers using Vector NTI software, except for the *βACT-1* gene, for which the primer sequences were obtained from a previous study in *E. multilocularis* [23] and already used in *E. granulosus* as reference gene [19]. For primer design, the following characteristics were considered: an amplification product between 100–200 bp, annealing temperature of 60±1°C, and location of the amplified sequence close to the 3′ end. The details of each selected gene and the characteristics of each primer are shown in Table 1 and Table 2, respectively. The qPCR reactions were performed using an ABI Real-Time 7500 PCR system (Applied Biosystems) with the following reaction mixture: 10 µL of diluted cDNA as template, 0.1× SYBR Green I (Invitrogen), 0.1 µM of each primer, 1× PCR Buffer (20 mM Tris-HCl pH 8.4, 50 mM KCl), 3 mM MgCl_2_, 25 µM dNTPs, 0.25 U Platinum Taq DNA polymerase, and MilliQ water in a final reaction volume of 20 µL (for each qPCR reagent, the final concentration is showed). A reverse transcription negative control (without reverse transcriptase) for each synthesized cDNA and a non-template negative control for each gene run were included to confirm the absence of genomic DNA and contamination of PCR reactions, respectively. The qPCR conditions were as follows: initial activation at 94°C for 5 min, followed by 40 cycles with denaturation at 94°C for 15 s, annealing at 60°C for 10 s and extension at 72°C for 35 s. A dissociation step from 94°C to 50°C with ramping increments of 0.1°C/s was added to assess the amplification specificity for each gene through melting curve analyses in SDS software (provided in ABI Real-Time 7500 system). The specificity of the amplified products was also analyzed by electrophoresis in 2% agarose gels. To determine the PCR amplification efficiency for each candidate to reference gene, standard cDNA dilutions were prepared using seven 2-fold serial dilutions. To calculate the PCR efficiency, the LinRegPCR software was used [24]. All qPCR reactions for each sample and each gene were performed in triplicate.

**Table 1:** Descriptions of candidate reference genes.

Gene symbol	Gene name	Function	aDatabases & Acc. number	bGeneDB Acc. Number
*βTUB*	Beta tubulin 2C chain	Cytoskeletal structural protein (microtubules)	LophDB EGC04893	EgrG_002026000
*βACT-1*	Beta actin-1	Cytoskeletal structural protein (microfilaments)	GenBank L07773	EgrG_000406900
*GAPDH*	Glyceraldehyde-3-phosphate dehydrogenase	Glycolytic enzyme	LophDB EGC00305	EgrG_000254600
*NDUFV2*	NADH dehydrogenase ubiquinone flavoprotein 2	Oxidoreductase activity	GeneDB	EgrG_001114700
*RPL13*	L13 ribosomal protein	Structural component of the large 60S ribosomal subunit	LophDB EGC01259	EgrG_000517800
*ETIF4A-III*	Eukaryotic translation initiation factor 4A-III	Translation	LophDB EGC00363	EgrG_001193600
*TBP*	TATA-Box binding protein	Transcription	GeneDB	EgrG_000972300
*CYP-1*	Cyclophilin	Protein folding and protein interactions	GenBank AF430707	EgrG_000920600
*RPII*	DNA directed RNA polymerase II subunit RPB2	Polymerization	GenBank FN566850	EgrG_000604200
*EF-1*α	Elongation Factor 1 alpha	Protein synthesis	GenBank AB306934.1	EgrG_000982200
*MAPK3*	Mitogen activated protein kinase 3	Signal transduction	GenBank HQ585923	EgrG_000803700

a Databases and accession numbers used in this work.

b Accession number obtained from GeneDB database after the *E. granulosus* genome annotation.

**Table 2:** Details of each primer designed for the candidate reference genes and target genes.

Gene symbol	Primer sequence (5′-3′) forward/reverse	Amplicon length (bp)	Tm	Amplification efficiency (%)
*βTUB*	CGTTCAGGCTACCGCCGGTT/GGAGCCCGTTGGGTCTACTCCGT	146	85.9	77.0
*βACT-1*	CGCGATCTCACCGACTGG/CTCCAGAGAGGAGCTAGTG	161	87.5	78.0
*GAPDH*	ACTCCGTCAATGTTGTCGCTGTCA/TAACCAACTTGCCGCCATCAACCT	128	84.0	92.1
*NDUFV2*	GACACCGCCATCAATAACAGGGAT/CCATTCTGCCGTTGTGCAATGT	146	85.3	85.2
*RPL13*	GAAGTGGCAATTCATGGTAAGGACG/CACAAGCAGGTTTGGGAGCGA	110	87.9	84.0
*ETIF4A-III*	AGTTCTCATTCTGTCGCCTACACGC/GACATTAGTGCCGCCATAGCAGG	115	85.4	82.4
*TBP*	TTCCAGCGCTCAGGCACACA/CGTGCGCTTTGAGCTATCCGTCT	165	86.5	87.5
*CYP-1*	CGACATCTCCATTGGCGGTAAGC/TTGTATCCGAAACCCTTCTCACCG	120	86.8	90.0
*RPII*	CATCTGCCGCCCGCTTGTTA/TCATGGCTGTCTCCTCCTCCAAAA	163	85.6	90.2
*EF-1*α	TTTGAGAAAGAGGCGGCTGAGATG/TAATAAAGTCACGATGACCGGGCG	174	87.9	92.0
*MAPK3*	AAAGTACAGCAGTTGAGTCGCGAGC/GCTTCAAATCTCGGTGCAAAACGTT	106	83.3	87.0
*RPL14*	TCCTTATCGATGGACCTTGTTCGG/TTCTGCCTGCGCCAATTATCCT	136	85.0	88.0^a^
*RPs15*	AATACGTCGAGGTCTCGGAACCAA/CAGGTGAGTTTTAACAACTGCCGGT	109	84.3	91.0^a^
*ELP*	GACACGCGATCAGTCGAAAATGC/TGTTGCCCTTGCGAATGTTGC	107	84.7	90.4^a^
*EgAgB1*	AAATGTTTGGCGAAGTGAAGT/ACCTGAGTGCCATGCGTAGCTTCT	126	84.5	90.0^a^^, ^ b
*EgAgB2*	AAAGCACACATGGGGCAAGTG/GTGTCCCGACGCATGACTTA	218	85.4	86.4^a^^, ^ b
*EgAgB3*	GAAGGGTGTGATGAAGGCCAT/ATACTCCTTCAGTGCCATGCGTGC	145	85.4	90.0^a^^, ^ b
*EgAgB4*	CGAGAGATGCAAGTGCCTCAT/GTGTCCCGACGCATGACTTA	219	86.0	86.0^a^^, ^ b
*EgAgB5*	GAAGATGACATCGATTCGAAA/GATCGAGCTTTTGTCCTGGC	155	83.4	N/C^b^^, ^ c

a Values correspond to the amplification efficiency average of the samples (both PS and PSP groups) used in the gene expression experiments and obtained with the LinRegPCR software.

b Amplification products confirmed by sequencing.

c N/C = not calculated.

### Gene expression stability analysis

geNorm [25] and NormFinder [26] are the two gene normalization algorithms generally used to analyze the expression stability of candidate reference genes. geNorm calculates the gene expression stability measure (M value) for a reference gene as the average pairwise variation (V) for that gene with all other tested reference genes. Moreover, geNorm determines an optimal number of reference genes for reliable normalization. NormFinder algorithm is based on the analysis of variance (ANOVA) mathematical model and allows the estimation of intra- and intergroup variation as well as the calculation of reference gene stability values. In addition, we used the RefFinder tool (http://www.leonxie.com/referencegene.php) for the assessment and screening of reference genes. The RefFinder tool integrates the currently available major computational programs (geNorm, Normfinder, BestKeeper, and the comparative ΔΔCt method) to compare and rank the tested candidate reference genes.

### Normalization of selected target genes

Once the most stably expressed genes were detected, we used the ΔΔCt method to quantify the expression of three selected target genes: two ribosomal proteins, L14 and s15, previously described as differentially expressed between *E. granulosus* PS and PSP conditions [10]; and the ezrin-radixin-moesin (ERM)-like protein (*ELP*) gene, described as constitutively expressed in PS and PSP conditions for *E. multilocularis* [20]. Also, we quantified the relative expression of the five genes that encode the widely studied *EgAgB1-5* genes. We used the available RNA-seq data [8] and previous qPCR analysis [19], [21] to discuss the gene expression of the different EgAgB subunits. The characteristics of the specific primers for these eight target genes (designed with Vector NTI software) are summarized in Table 2. Using the 2^−ΔΔCT^ values, we compared the PS and PSP experimental groups through the paired samples *t* test. Furthermore, the differences between the relative quantities of each *EgAgB1-5* gene were assessed by ANOVA. Statistical analyses were performed using SPSS software. In this assay, three biological replicates and two technical replicates were used.

## Results

A total of 10 cysts were collected, with 3 belonging to *E. granulosus* s.s. (G1) and 7 belonging to *E. ortleppi*. We used paired PS and PSP samples for all *E. granulosus* s.s. (G1) and for five *E. ortleppi* cysts. For the other two *E. ortleppi* cysts, only a PS or PSP sample (unpaired samples) was analyzed due to the low amount of parasite material. Neither genomic DNA nor RNA degradation was observed for any of the total RNA samples analyzed (Figure S1). As expected for several Platyhelminthes species [27], a single band of total RNA was observed on the agarose gel and in the Bioanalyzer analysis in all samples (Figure S1).

### qPCR

**Figure 1:**
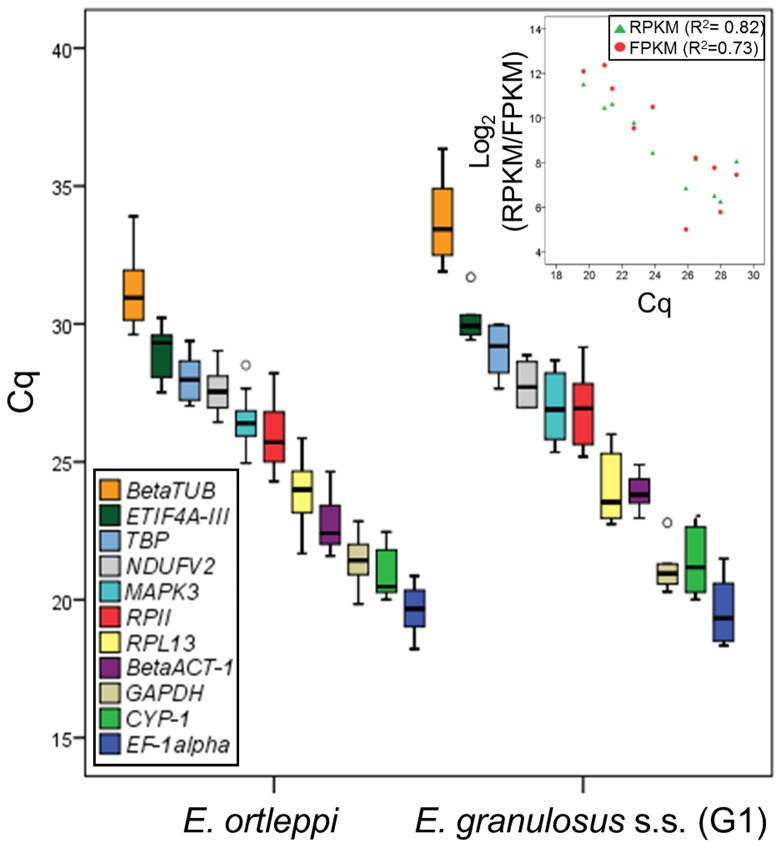
Assessment of transcript abundance and comparison with RNA-seq data. Note the similarity in the boxplot distribution for each gene and the high dispersal values for several genes, such as *βTUB* and *RPII*, in both species. The mean Cq value for each gene (including both PS and PSP experimental groups) in *E. ortleppi* was compared with the published RNA-seq data for *E. granulosus* spp. protoscolex (FPKM from the Cuffdiff program [9]; RPKM from the ERANGE program [8]) as shown in the top right. Due to a high standard deviation, the *βTUB* gene was excluded in the qPCR vs. RNA-seq comparison.

For all PCR products, we did not detect amplification of nonspecific products, formation of primer dimers or any PCR contaminants (Figure S2). The amplification efficiency for each candidate to reference gene and for each target gene is shown in Table 2. For the *EgAgB5* gene the amplification efficiency value were not calculated due to its very low expression level, avoiding the formation of the *plateau*, and thus, the amplification efficiency calculation by the LinRegPCR software. The amplification curves used to calculate the amplification efficiency of the candidate reference genes are shown in Figure S3. Once all the cycle quantification (Cq) values of the qPCR were obtained, we assessed the transcript abundance of each gene in *E. granulosus* s.s. (G1) and *E. ortleppi* (Figure 1). In both species, the transcript distribution was almost the same, which was expected for closely related species and apparent housekeeping genes. Moreover, we found that *βTUB*, *RPL13*, *RPII* and *βACT-1* contain the most dispersal Cq values in *E. ortleppi*, while in *E. granulosus* s.s. (G1) it is observed for *βTUB* and *RPII*. Furthermore, because of the greater number of biological replicates for *E. ortleppi*, we based on the mean of both PS and PSP values of each gene to compare our qPCR data versus the RNA-seq data for *E. granulosus* spp. protoscolex (expressed as Reads Per Kilobase per Million (RPKM) [8] and Fragments Per Kilobase per Million (FPKM) [9]). A linear correlation (R^2^) of 0.67 and 0.56 in *E. granulosus* s.s. (G1) and 0.82 and 0.73 in *E. ortleppi* was observed for RPKM and FPKM, respectively (Figure 1, inset).

### Gene expression stability analysis

**Figure 2:**
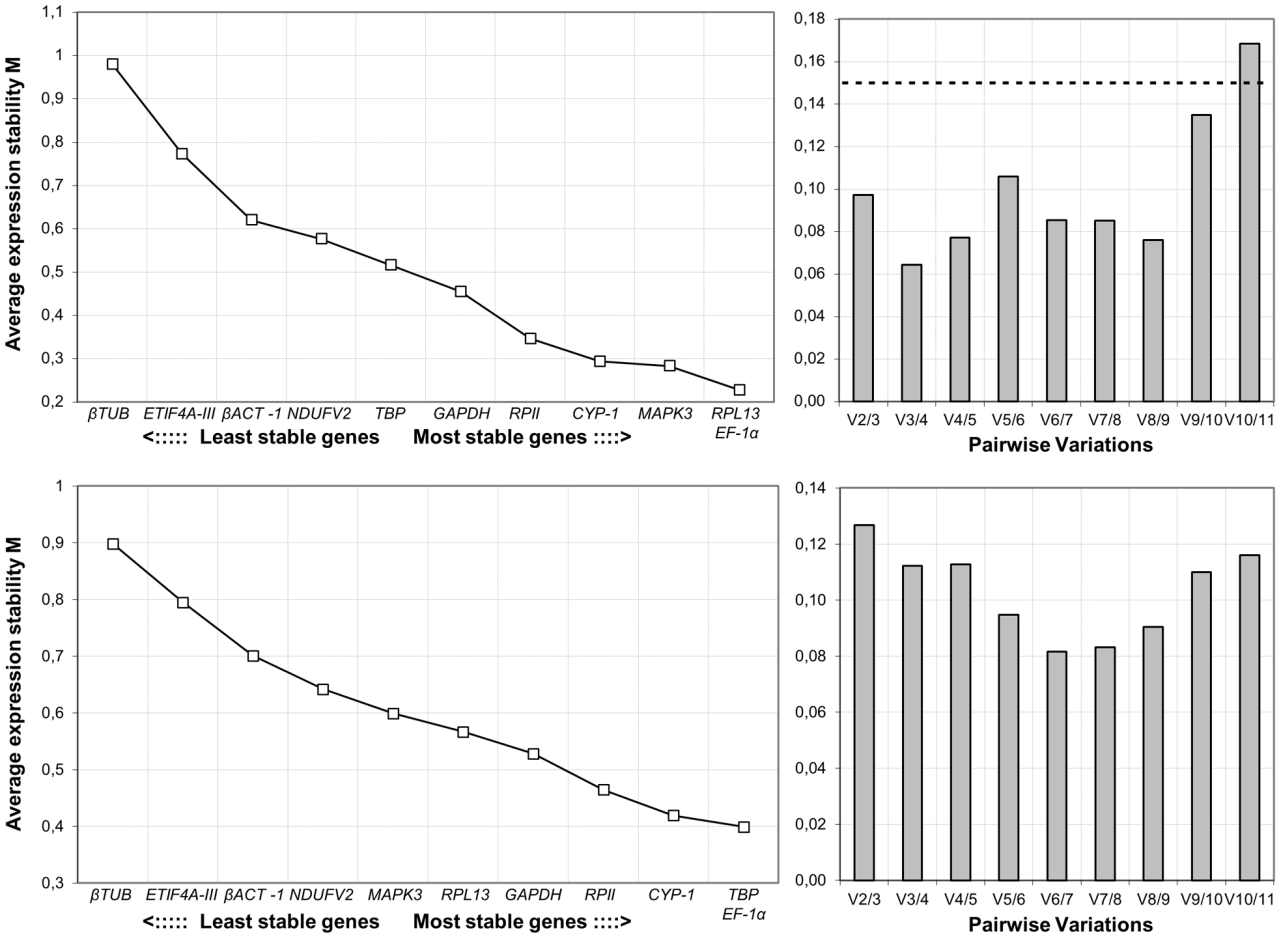
Gene expression stability (left) and determination of the optimal number of reference genes (right) by geNorm. The top charts correspond to *E. granulosus* s.s. (G1), and the bottom charts correspond to *E. ortleppi*. The dotted line represents the cut-off of 0.15 proposed by geNorm authors to determine the optimal number of reference genes to be used.

The most common algorithms available to assess gene expression stability were employed, and the results were compared [25], [26]. These algorithms required the transformation of Cq values to relative quantities. Thus, the mean Cq values from triplicate runs were converted into relative quantities by the ΔΔCt method and used as input data for both the geNorm and NormFinder algorithms. Figure 2 shows the two output charts from the geNorm program for each species. Regarding the average expression stability values, *EF-1α* and *TBP* were the most stable genes in *E. ortleppi*, and *EF-1α* and *RPL13* were the most stable genes in *E. granulosus* s.s. (G1). In contrast, *βTUB*, *ETIF4A-III*, *βACT-1* and *NDUFV2* were the least stables genes for both species. The other geNorm chart showed the optimal number of reference genes that would be necessary for suitable gene expression normalization. In both species, the V2/3 values were less than 0.15 (the cut-off value recommended by geNorm authors), thereby indicating that the optimal number of reference genes for normalization is 2. The other algorithm that allows the identification of the most reliable reference genes from a set of candidate genes is Normfinder. Here, the ranking and stability values obtained for each gene (and also in geNorm) were exactly the same as those obtained by RefFinder. For this reason, we plotted only the RefFinder output data in Table 3. The comprehensive ranking for *E. ortleppi* was similar to that of *E. granulosus* s.s. (G1). However, the ordinal order given by each gene expression stability algorithm was more variable in *E. granulosus* s.s. (G1) than in *E. ortleppi*. Performing random samplings of the Cq values for 3 paired samples of *E. ortleppi* and by placing these data as input in RefFinder, we corroborated that the differences in the ordinal order obtained was clearly a consequence of the number of biological replicates used (Table S1A, S1B and S1C). For *E. ortleppi*, *TBP*, *EF-1α* and *CYP-1* were the most stable genes, and *ETIF4A-III* and *βTUB* were the least stable genes (Table 3). For *E. granulosus* s.s. (G1), *TBP*, *EF-1α* and *GAPDH* were the most stables genes, and *RPII* and *βTUB* were the least stable genes (Table 3). Interestingly, for both species, none of the genes described as housekeeping genes in the *Echinococcus* literature (*βACT-1*, *ETIF4A-III*, *CYP-1* and *MAPK3*) demonstrated a good expression stability value (Figure 2).

**Table 3:** Gene expression stability for *E. granulosus* s.s. (G1) (top) and *E. ortleppi* (bottom) as assessed by RefFinder.

Ranking Order (Better→Good→Average)											
Method	1	2	3	4	5	6	7	8	9	10	11
Delta CT	TBP	EF-1α	GAPDH	MAPK3	CYP-1	RPII	βACT-1	NDUFV2	RPL13	ETIF4A-III	βTUB
BestKeeper	ETIF4A-III	βACT-1	GAPDH	TBP	NDUFV2	CYP-1	EF-1α	MAPK3	RPL13	RPII	βTUB
Normfinder	TBP	GAPDH	βACT-1	NDUFV2	EF-1α	MAPK3	CYP-1	RPII	RPL13	ETIF4A-III	βTUB
geNorm	RPL13 | EF-1α		MAPK3	CYP-1	RPII	GAPDH	TBP	NDUFV2	βACT-1	ETIF4A-III	βTUB
**Recommended comprehensive ranking**	**TBP**	**EF-1α**	**GAPDH**	**βACT-1**	**MAPK3**	**RPL13**	**CYP-1**	**ETIF4A-III**	**NDUFV2**	**RPII**	**βTUB**

Ranking Order (Better→Good→Average)											
Method	1	2	3	4	5	6	7	8	9	10	11
Delta CT	TBP	EF-1α	CYP-1	RPII	NDUFV2	MAPK3	GAPDH	βACT-1	RPL13	ETIF4A-III	βTUB
BestKeeper	TBP	EF-1α	CYP-1	GAPDH	MAPK3	NDUFV2	RPII	βACT-1	RPL13	βTUB	ETIF4A-III
Normfinder	TBP	EF-1α	CYP-1	NDUFV2	RPII	MAPK3	βACT-1	GAPDH	RPL13	ETIF4A-III	βTUB
geNorm	TBP | EF-1α		CYP-1	RPII	GAPDH	RPL13	MAPK3	NDUFV2	βACT-1	ETIF4A-III	βTUB
**Recommended comprehensive ranking**	**TBP**	**EF-1α**	**CYP-1**	**RPII**	**NDUFV2**	**GAPDH**	**MAPK3**	**βACT-1**	**RPL13**	**ETIF4A-III**	**βTUB**

doi:10.1371/journal.pone.0102228.t003

### Relative Quantification Of The Selected Target Genes

**Figure 3:**
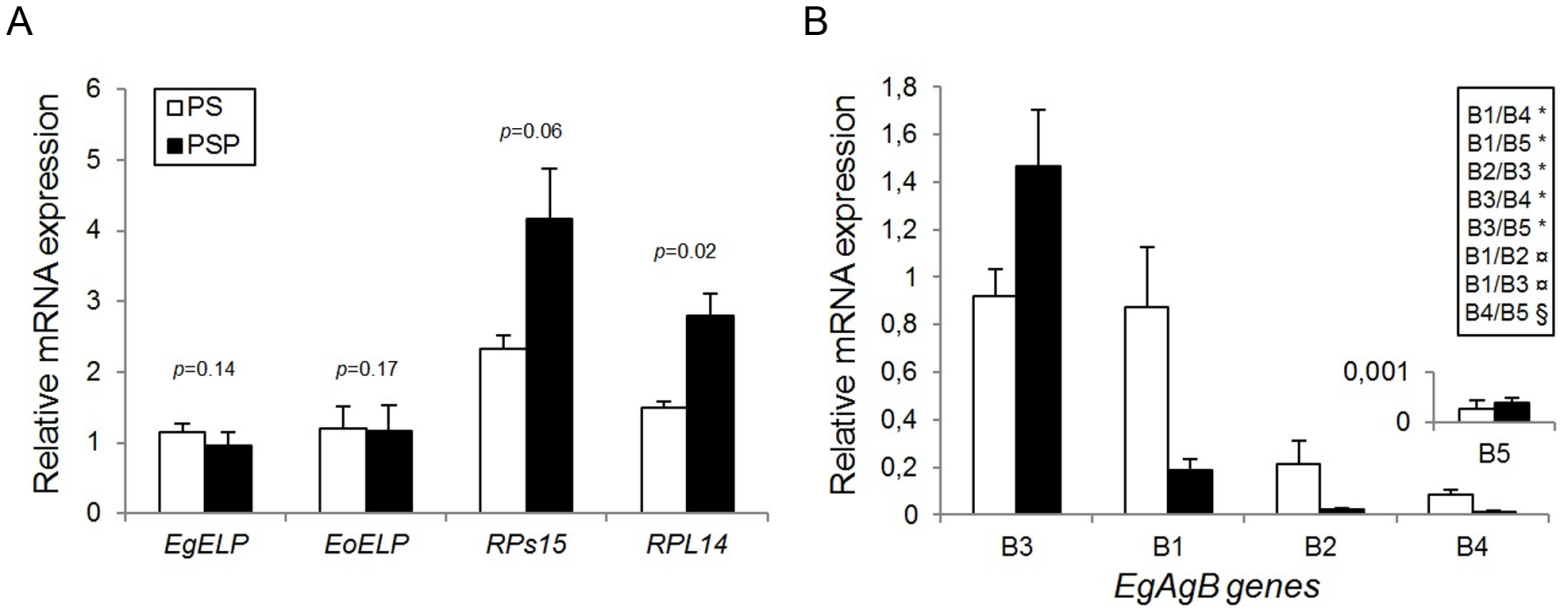
Relative mRNA expression of the selected target genes using *EF-1α* as reference gene. The constitutive (*ELP*) and differential (*RPs15* and *RPL14*) gene expression between both PS and PSP conditions are shown in (A). Here, the PS treatment of the *EgELP* gene was used as calibrator to obtain the 2^−ΔΔCT^ values and, subsequently, the bar graph bar. *E. ortleppi* was used only for the *ELP* gene (*EoELP*) expression analysis, and *E. granulosus* was used to analyze the *ELP* gene (*EgELP*) and all other target genes. Results for the *EgAgB1-5* genes (B) are consistent with previous works in *Echinococcus* spp. [8],[21], where *EgAgB1* and *EgAgB3* are the most abundant in protoscoleces, and *EgAgB5* exhibit a very low gene expression. The increase of *EgAgB1* and the decline of *EgAgB2*, *EgAgB3* and *EgAgB4* genes in the PSP group is comparable to previously described for the immature adult form of *E. multilocularis* [21]. The statistical significance between EgAgB subunits in both PS and PSP groups (*) as well as only for PSP (¤) or only for PS (§) are shown in the inset. Here, the PS treatment of the EgAgB3 subunit was used as a calibrator. A significance level of α = 0.05 was considered.

Firstly, with the most stable gene identified, we performed a relative quantification analysis of three selected target genes, the *RPL14*, the *RPs15* and the *ELP* genes. Significant statistical differences was solely observed for the *RPL14* gene (p<0.05) (Figure 3A). The *RPs15* gene showed a clear tendency to increase in the PSP experimental condition, with p = 0.06, in contrast to the *ELP* gene, which exhibit no significant difference for both *E. granulosus* s.s. (G1) and *E. ortleppi* (p values of 0.14 and 0.17, respectively) (Figure 3A). Based on these consistent results with the previously described [10],[20], we normalized the *EgAgB1-5* gene expression using *EF-1α* as the reference gene. The significant differences between each *EgAgB1-5* gene are shown in Figure 3B (inset). As described previously [19],[21], *EgAgB3* was the most abundant, and *EgAgB5* was expressed at a low level. *EgAgB1* showed a moderate expression level, followed by *EgAgB2*, *EgAgB4* and *EgAgB5*. Among the PS and PSP experimental groups, *EgAgB1*, *EgAgB2*, and *EgAgB4* showed considerable variation as indicated by the bar graph (Figure 3) and qPCR curves (ΔCq variation of 0.6–3.35 for *EgAgB1*, 1.66–3.57 for *EgAgB2* and 0.86–4.25 for *EgAgB4*), but no significant differences were found for all *EgAgB1-5* genes.

## Discussion

Faced with a vast amount of gene expression data, it is important to recognize and understand which genes are upregulated or downregulated as well as which genes are specific to a particular life cycle stage. The elucidation of the dynamic behavior of gene expression is also expected to provide new insights into parasite development and host-parasite relationships. Recently, a large amount of sequencing (both genomic and transcriptomic) data for *Echinococcus* genus and other related parasites was published and made available in public databases [8],[9]. However, there are very few publications that validate the transcriptomic data. A more comprehensive overview considering the available protein expression data for *E. granulosus* [28],[29] could help to obtain and understand the correlations between transcription and translation pathways. In our analysis, we focused on the identification of genes that are stably expressed (normalizer or reference genes) in PS and PSP experimental conditions of *E. granulosus* s.s. (G1) and *E. ortleppi*. In addition to confirming the transcriptomic data of several selected genes, we showed a suitable and reliable validation of gene expression stability for *Echinococcus* spp. in the initial stage of the pre-adult development.

We selected eleven candidates for reference genes, seven of which were usually employed in previous studies (*βTUB*, *NDUFV2*, *RPL13*, *TBP*, *CYP-1*, *RPII*, and *EF-1α*) and four of which were previously identified as housekeeping genes in *Echinococcus* spp. (*βACT-1*, *GAPDH*, *ETIF4A-III*, and *MAPK3*). Another criterion for selecting candidate reference genes was that the genes were involved in different functions or pathways (metabolism, structural, translation, and signal transduction) to avoid a possible co-regulation between selected genes. Despite the lack of validated reference genes, several publications involving gene expression analysis on *Echinococcus* spp. have been published [16]–[19],[30]–[33]. The *βACT-1* gene is widely used as a housekeeping gene, but this gene has been shown to be significantly upregulated in immature adult worms and to be variable in the other stages. Thus, the translation initiation factor of *E. granulosus* (*Eg-eif*) was proposed as an alternative housekeeping gene [19]. Importantly, this previous study showed that one of the most commonly used genes is not a good normalizer for gene expression analysis. However, the gene proposed by the authors was not validated, thereby creating a new uncertainty regarding reliable gene expression quantification. Other genes identified with apparently constitutive expression in *E. granulosus* were cyclophilin in protoscoleces [14] and the extracellular signal-regulated kinase in the cyst wall and protoscoleces [15]. Although the selection of the candidate reference genes was not based on recently published RNA-seq quantification data for *Echinococcus* spp., we identified stably expressed genes to be used as reference genes. It is worth noting that our qPCR data for several selected genes confirmed and validated those obtained from transcriptome analysis of *E. granulosus* spp. [8],[9] (Figure 1) and from other studies where *CYP-1*, *GAPDH* and *βACT-1* generally showed high transcript abundance [34]–[36].

Several different algorithms are available to identify the relative stability of genes from a given set of candidate reference genes. Generally, these algorithms show a stability value and an ordinal ranking that allow selection of the best reference gene for further analysis of gene expression quantification. geNorm was the first program to be published [25], and it is currently the most used to identify normalizer genes. In addition to the stability value, geNorm gives the number of reference genes that would be necessary for suitable gene expression normalization. For *E. granulosus* s.s. (G1), *EF-1α* and *RPL13* were the recommended reference genes. However, when we compared the geNorm ranking with those generated by other programs (Table 3), we found that *TBP* and *EF-1α* were the most stable genes and that *RPL13* was not included within the most stable genes. Similar results were obtained for *E. ortleppi*, where the distribution of the most and least stables genes for each method was correlated (Table 3), which may have been due to the number of samples used in this species. The difference in the number of biological samples was due to differences in the species frequency in the region sampled, as discussed previously [37]. For *E. ortleppi*, *TBP* and *EF-1α* were the most stable genes, which was the same as the RefFinder ranking observed for *E. granulosus* s.s. (G1). An interesting observation in the *E. granulosus* s.s. (G1) RefFinder ranking was that *TBP* was the most stable gene regarding the comparative ΔΔCt and NormFinder methods but not for the geNorm program, suggesting that the comparative analysis performed by RefFinder is an efficient strategy to determine a suitable ranking from a set of candidate reference genes, mainly when the number of replicates is small. Based on this comparative analysis between the two related species, we propose the use of *EF-1α* and *TBP* genes as reference genes for studies that involve gene expression analysis of *E. granulosus* s.s. (G1) and *E. ortleppi* PS and PSP experimental conditions. In accordance with the similar results obtained for each species, we suggest that *EF-1α* and *TBP* could be used to normalize the gene expression in studies involving other *E. granulosus* s.l. species. It is important to note that we focused on the early pre-adult development of *Echinococcus* spp., excluding the germinal layer tissue and other parasite life cycle stages, such as the onchosphere or the adult form. Thus, for gene expression quantification studies including these stages, new reference genes should be validated.

Finally, we performed a relative quantification analysis of different selected target genes. At first, studying constitutive and differentially expressed genes between the PS and PSP conditions in, we showed that consistent data are obtained when the most stable *EF-1α* gene is used as normalizer. Based in these results, we measure the expression levels of the*EgAgB1-5* genes using *EF-1α* as a reference gene in both PS and PSP experimental groups for *E. granulosus*. The results for *EgAgB1*, *EgAgB3* and *EgAgB5* were consistent with RNA-seq data [8]. *EgAgB3* was the most abundant, followed by *EgAgB1* with a moderate gene expression level and *EgAgB5* displaying a low gene expression level in PS samples but a slightly increased expression level in PSP samples. Previous proteomic analyses have detected the presence of the EgAgB1 subunit in protoscoleces [28] as well as in the hydatid fluid and germinal layer [29]. Our results were also consistent with the *EgAgB1-5* expression data where *CYP-1* was used as a reference gene in *E. multilocularis* [21]. Although *CYP-1* was not the most stable gene in our analysis, its expression stability remained among the most stable, thus leading to analogous results. Based on these findings, we show that *EF-1α* can be reliably used as a reference gene in expression studies involving protoscoleces and/or pepsin-treated protoscoleces.

In this work, we focused on the early pre-adult development of *Echinococcus* spp. that occurs in the most crucial stage of the parasite life cycle, the metacestode stage, which can accidentally occur in humans causing CE or AE. We identified that *EF-1α* is a suitable and reliable reference gene for gene expression normalization, both in protoscoleces and their pepsin “activated” stage obtained experimentally *in vitro*. This report validates suitable reference genes for gene expression studies in two species of the class Cestoda, phylum Platyhelminthes, and provides a basis for further analysis in other species, such as those that cause Taeniasis, another important neglected tropical disease as indicated by WHO.

## Supporting Information

Figure S1 Total RNA isolation. In all samples a single band of total RNA was observed on the 1.5% agarose gel (left) and in the Bioanalyzer analysis (right). Total RNA extraction also displays absence of genomic DNA and RNA degradation. Eg refers to *E. granulosus* and Eo to *E. ortleppi*.
(TIF)

Figure S2 Amplification specificity of the primers. A single band was observed for each amplicon in the 2% agarose gel (top). The melting curves obtained for each gene (below the agarose gel) also show a specific curve without any contaminants.
(TIF)

Figure S3 Amplification curves used to calculate the amplification efficiency for each selected gene. Numbers from 1 to 7 (in red color) indicate the dilutions that were used for the amplification efficiency calculation of the candidate reference genes.
(TIF)

Table S1 RefFinder output tables for *E. ortleppi*. Based on three random samplings of 3 paired samples of *E. ortleppi*, the results obtained (A, B and C) were similar to those in the *E. granulosus* s.s. (G1) RefFinder output in Table 3.
(DOCX)
