# Supplementary figures and images for: Validation of Suitable Reference Genes for Expression Normalization in Echinococcus spp. Larval Stages
Source: PLoS One. 2014 Jul 11;9(7):e102228. doi: 10.1371/journal.pone.0102228 (PMC4094502; doi:10.1371/journal.pone.0102228)

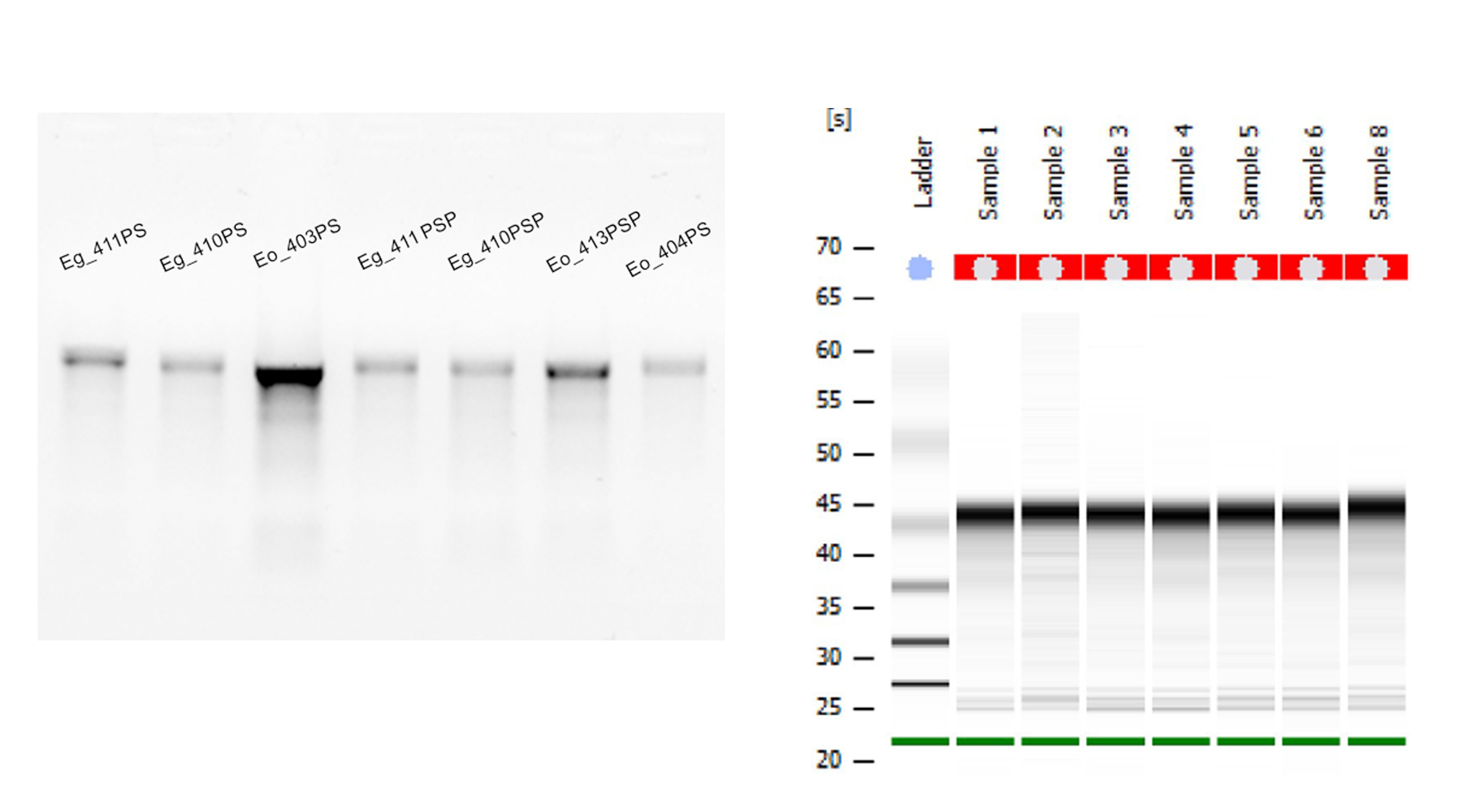

Supplement: Figure S1 — In all samples a single band of total RNA was observed on the 1.5% agarose gel (left) and in the Bioanalyzer analysis (right). Total RNA extraction also displays absence of genomic DNA and RNA degradation. Eg refers to E. granulosus and Eo to E. ortleppi. (TIF) [file pone.0102228.s001.tif]

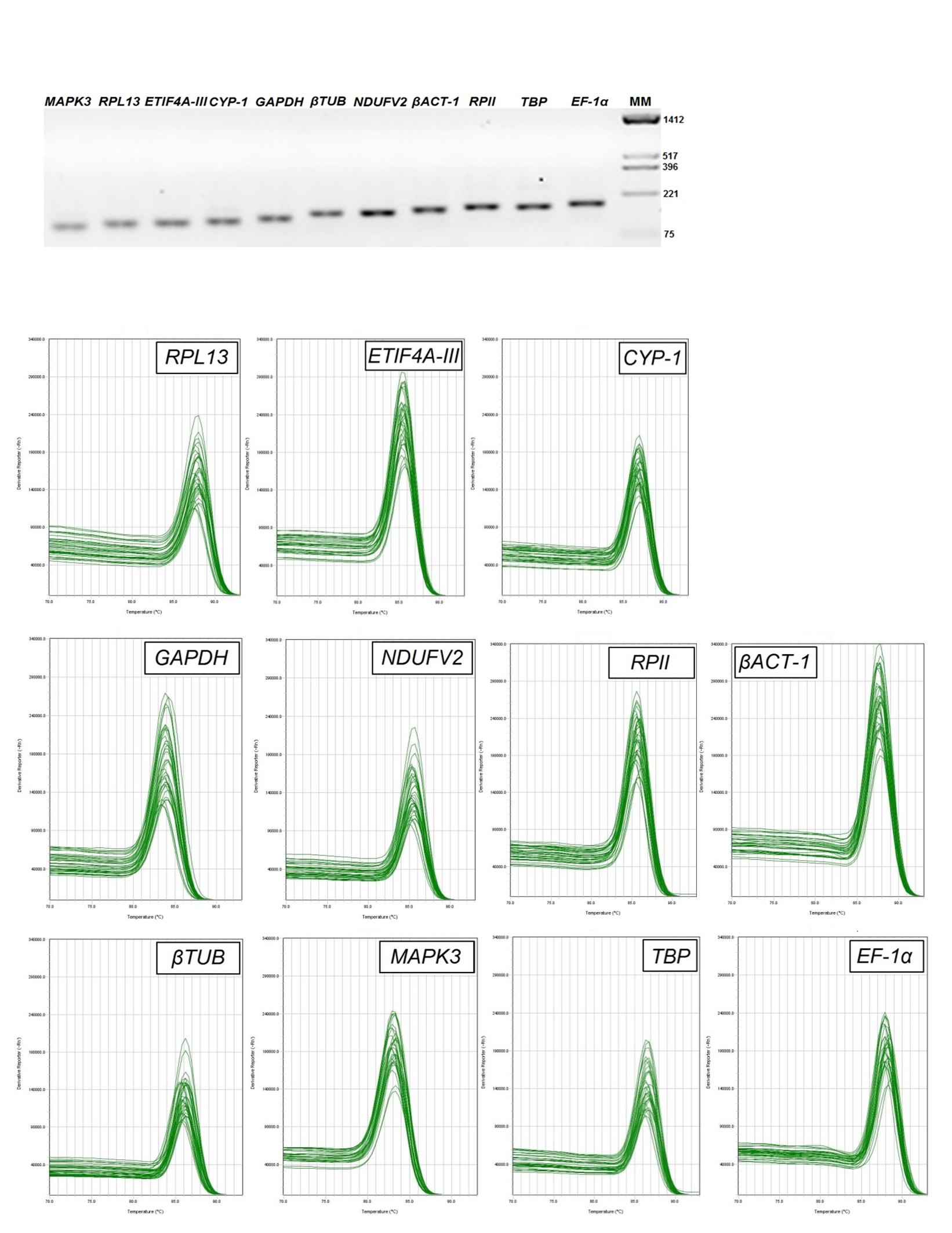

Supplement: Figure S2 — A single band was observed for each amplicon in the 2% agarose gel (top). The melting curves obtained for each gene (below the agarose gel) also show a specific curve without any contaminants. (TIF) [file pone.0102228.s002.tif]

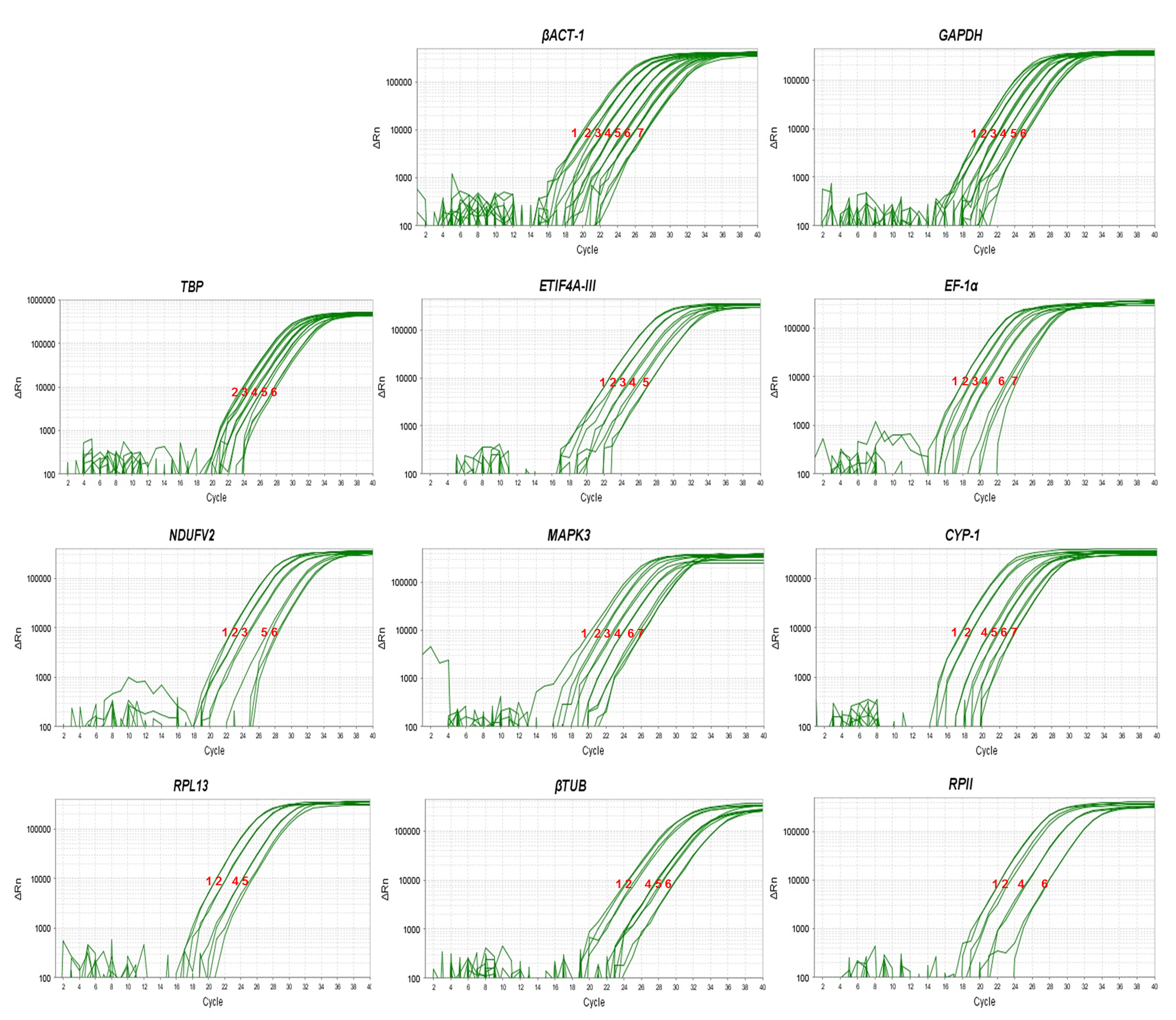

Supplement: Figure S3 — Numbers from 1 to 7 (in red color) indicate the dilutions that were used for the amplification efficiency calculation of the candidate reference genes. (TIF) [file pone.0102228.s003.tif]
