# Supplementary material for: Validation of Suitable Reference Genes for Expression Normalization in Echinococcus spp. Larval Stages
Source: PLoS One. 2014 Jul 11;9(7):e102228. doi: 10.1371/journal.pone.0102228 (PMC4094502; doi:10.1371/journal.pone.0102228)
Supplement: Table S1 — Based on three random samplings of 3 paired samples of E. ortleppi, the results obtained (A, B and C) were similar to those in the E. granulosus s.s. (G1) RefFinder output in Table 3. (DOCX) [file pone.0102228.s004.docx]

**Table S1.** RefFinder outputs tables for *E. ortleppi*. Based on three random sampling of 3 paired samples of *E. ortleppi* is possible to obtain a similar result (A, B and C) when compared with the *E. granulosus* s.s. (G1) RefFinder output on Table 3.

**A**

| **Ranking Order (Better🡪Good🡪Average)** | | | | | | | | | |  |  |
| --- | --- | --- | --- | --- | --- | --- | --- | --- | --- | --- | --- |
| **Method** | 1 | 2 | 3 | 4 | 5 | 6 | 7 | 8 | 9 | 10 | 11 |
| Delta CT | RPL13 | CYP-1 | NDUFV2 | βTUB | EF-1α | ETIF4A-III | TBP | GAPDH | MAPK3 | βACT -1 | RPII |
| BestKeeper | GAPDH | MAPK3 | EF-1α | βTUB | NDUFV2 | TBP | CYP-1 | ETIF4A-III | RPL13 | βACT-1 | RPII |
| Normfinder | RPL13 | CYP-1 | NDUFV2 | βTUB | EF-1α | ETIF4A-III | TBP | GAPDH | MAPK3 | βACT-1 | RPII |
| Genorm | NDUFV2\| RPL13 | | CYP-1 | βTUB | EF-1α | ETIF4A-III | TBP | GAPDH | MAPK3 | βACT-1 | RPII |
| **Recommended comprehensive ranking** | **RPL13** | **NDUFV2** | **CYP-1** | **βTUB** | **EF-1α** | **GAPDH** | **MAPK3** | **ETIF4A-III** | **TBP** | **βACT-1** | **RPII** |

**B**

| **Ranking Order (Better🡪Good🡪Average)** | | | | | | | | | | |  |  |
| --- | --- | --- | --- | --- | --- | --- | --- | --- | --- | --- | --- | --- |
| **Method** | 1 | 2 | 3 | 4 | 5 | 6 | 7 | 8 | 9 | 10 | | 11 |
| Delta CT | TBP | CYP-1 | RPII | EF-1α | NDUFV2 | MAPK3 | βACT-1 | GAPDH | RPL13 | ETIF4A-III | | βTUB |
| BestKeeper | CYP-1 | βACT-1 | EF-1α | TBP | NDUFV2 | MAPK3 | RPII | GAPDH | RPL13 | ETIF4A-III | | βTUB |
| Normfinder | TBP | RPII | NDUFV2 | CYP-1 | EF-1α | βACT-1 | MAPK3 | GAPDH | RPL13 | ETIF4A-III | | βTUB |
| Genorm | CYP-1 \| EF-1α | | TBP | RPII | MAPK3 | NDUFV2 | GAPDH | RPL13 | βACT-1 | ETIF4A-III | | βTUB |
| **Recommended comprehensive ranking** | **CYP-1** | **TBP** | **EF-1α** | **RPII** | **NDUFV2** | **βACT-1** | **MAPK3** | **GAPDH** | **RPL13** | **ETIF4A-III** | | **βTUB** |

**C**

| **Ranking Order (Better🡪Good🡪Average)** | | | | | | | | | | | | | | | | | |  |  |  |  |
| --- | --- | --- | --- | --- | --- | --- | --- | --- | --- | --- | --- | --- | --- | --- | --- | --- | --- | --- | --- | --- | --- |
| **Method** | 1 | 2 | 3 | 4 | | 5 | | 6 | | 7 | | 8 | | 9 | | 10 | | | 11 | |  |
| Delta CT | EF-1α | NDUFV2 | GAPDH | RPII | | TBP | | RPL13 | | CYP-1 | | MAPK3 | | βACT-1 | | βTUB | | | ETIF4A-III | |  |
| BestKeeper | NDUFV2 | EF-1α | GAPDH | TBP | | MAPK3 | | RPL13 | | CYP-1 | | RPII | | TUB | | ETIF4 | | | βACT-1 | |  |
| Normfinder | EF-1α | NDUFV2 | RPII | TBP | | GAPDH | | MAPK3 | | RPL13 | | CYP-1 | | βACT-1 | | βTUB | | | ETIF4A-III | |  |
| Genorm | CYP-1 \| RPL13 | | GAPDH | | TBP | | RPII | | EF-1α | | NDUFV2 | | MAPK3 | | βACT-1 | | βTUB | | | ETIF4A-III | |
| **Recommended comprehensive ranking** | **EF-1α** | **NDUFV2** | **GAPDH** | **RPL13** | | **TBP** | | **CYP-1** | | **RPII** | | **MAPK3** | | **βACT-1** | | **βTUB** | | | **ETIF4A-III** | |  |
